# Synthesis of optimized propolis solid lipid nanoparticles with desirable antimicrobial, antioxidant, and anti-cancer properties

**DOI:** 10.1038/s41598-023-45768-y

**Published:** 2023-10-25

**Authors:** Fatemeh Shahab-Navaei, Ahmad Asoodeh

**Affiliations:** 1 Department of Chemistry, Faculty of Science, Ferdowsi University of Mashhad, Mashhad, Iran; 2 Cellular and Molecular Research Group, Institute of Biotechnology, Ferdowsi University of Mashhad, Mashhad, Iran

**Keywords:** Biochemistry, Biotechnology

## Abstract

This study aimed to produce stable propolis nanoparticles with a size below 100 nm, suitable for various applications in industries such as pharmaceuticals, medicine, cosmetics, food, and packaging. To achieve this, propolis solid lipid nanoparticles (PSLNs) were synthesized using the hot homogenization method, and the optimized nanoparticles were analyzed using Design Expert software. The properties of the synthesized PSLN were characterized using UV–visible spectroscopy, FTIR, XRD, PSA, TEM, and zeta potential analysis. The results indicated that PSLNs with a size range of 57 ± 15 nm remained stable in an aqueous medium at pH 7.4. HPLC analysis showed that the active ingredient of phenols and flavonoids in the extract remained stable after the formation of PSLNs. Antioxidant and antibacterial properties of the extract and nanoparticles were also evaluated. The results demonstrated that the biological properties of the extract were effectively preserved in PSLNs, Additionally, the PSLN synthesized exhibited remarkable anticancer properties against the A549 cell line and with IC_50_ of 0.01 mg/ml after 72 h-treatment. In conclusion, the optimized PSLNs can be utilized as antioxidant and antibacterial additives and have the potential to be used as a drug or drug carrier for the treatment of lung cancer.

## Introduction

Propolis is one of honey bees’ most widely used products^1^. It acts as a defensive barrier against external factors^2^. This material is dense and viscous, similar to that of beeswax. The color of it ranges from yellow to dark brown^3^. Propolis does not have a fixed melting point, but it typically melts at 65 ℃. Over 300 bioactive compounds have been identified in propolis so far^4^. Phenolic compounds, esters, flavonoids, terpenes, steroids, aromatic aldehydes, and alcohols are among the most significant chemical compounds^5^. Propolis collected from various regions contains a variety of enzymes, including acid phosphatases, and vitamins such as B, C, and E^6^.

The long-term consumption of raw propolis may cause allergic reactions^7^. Only 15% of the beneficial propolis ingredients are absorbed by the body when consumed in its raw form. So far, many studies have reported the safety of propolis for humans and mice. Of course, it should be taken into account that these reports refer to the use of this substance in the prescribed standard doses. It should be noted that using this substance in high doses without a prescription can be dangerous^8^. Among the biological properties of propolis, antibacterial, antifungal, antiparasitic, antiviral, anticancer, and antioxidant properties have been reported^9–13^.

Instability in an aqueous medium and its low absorption rate in the body are some of the main issues associated with the use of propolis in its raw form or as extracts. These problems have posed challenges in the biomedical applications of Propolis^14^. One viable solution for extracting active compounds from Propolis and enhancing their stability in an aqueous medium is to create nanoparticles of Propolis. On the other hand, the production of Propolis nanoparticles can improve the cellular uptake of this substance, making it easier to use in pharmaceutical applications^14^.

Various methods have been reported for the preparation of Propolis nanostructures, with the most important being solid lipid nanoparticles (SLNs) due to the presence of lipid compounds in the Propolis structure^15^. SLNs are colloidal carriers ranging from 50 to 1000 nm, composed of lipids dispersed in the aqueous phase^16^.

Many researchers have synthesized propolis micro or nanoparticles greater than 100 nm^17–21^. However, the goal of this research is to synthesize propolis nanoparticles with a size of less than 100 nm in a stable manner in aquatic environments. This will allow for efficient use in medical, pharmaceutical, cosmetics, food, and packaging industries.

In this study, propolis was collected from beehives situated in the Binalood Mountain range (Latitude 36°19′12″N 59°13′28″E), and an ethanolic extract was prepared using the Soxhlet method. The ethanolic extract of propolis contains a considerable amount of insoluble resinous compounds that can lead to complications when combined with water. We used the lipid compound present in Propolis to produce solid lipid nanoparticles (SLN) through the hot homogenization method^22^.

The optimization of PSLNs was performed using Design Expert software^23^. The physicochemical characteristics of solid lipid nanoparticles (SLNs) were investigated through various analyses. Finally, to assess the quality of the yield, several biological tests were conducted on PSLNs and the propolis ethanolic extract (PEE). The results have been analyzed and compared.

## Experimental

### Materials

Crude propolis was collected from Binalood Mountain in Khorasan Razavi Province (Latitude 36°19′12″N 59°13′28″E). Ethanol from Merck was used as the solvent for extraction. Tween-20 (Merck) and Tween-80 (Merck) were used as surfactants. Phosphotungstic acid was purchased from Merck and utilized for negative staining purposes. Pure salicylic acid, parahydroxybenzoic acid, vanillic acid, gallic acid, and aluminum chloride were purchased from Merck. Deionized water was utilized in all of the experiments.

### Bacterial culture

Gram-positive bacteria, including *Staphylococcus aureus* (ATCC 25,923) and *Bacillus subtilis* (ATCC 6051), as well as Gram-negative bacteria, such as *Escherichia coli* (ATCC 25,923) and *Pseudomonas aeruginosa* (ATCC 9027), were utilized to investigate the antimicrobial properties of the compounds.

### Cell culture

The A549 cell line was utilized in this study. A549 is a malignant cell line derived from human pulmonary epithelial carcinoma.

### Propolis ethanolic extraction (PEE)

Propolis ethanolic extract (PEE) was prepared using a Soxhlet apparatus with a volume of 250 mL^24^. To extract propolis, 2.5 g of it were subjected to liquid nitrogen for 2 min to make it brittle, and then it was crushed into small fragments. The resulting dry powder was placed onto a filter paper and introduced into the Soxhlet reservoir. Next, 250 mL of 96% ethanol was added to the balloon of the Soxhlet apparatus, which was then placed in an oil bath and heated for 8 h at a temperature of 70 °C. The solvent was removed by transferring the ethanolic mixture to a rotary evaporator and rotating it at 100 rpm for 30 min at a constant temperature of 70 °C.

Equation (1) was used to calculate the amount of extract in this stage:

1 $$ Propolis \, Extraction \,  Efficency\left( \% \right) = \frac{{m_{t} - m_{S} }}{{m_{t} }} \times 100 $$

In the equation above, "*m*_*t*_" represents the total amount of propolis, while "*m*_*s*_" represents the amount of propolis that remains on the filter paper.

After Soxhlet extraction, the ethanolic extract was subjected to rotary evaporation to remove the solvent. The solvent was removed, and then the entire extract was transferred to a freeze dryer for drying. The final solution was stored in a closed container at −4 °C for use in nanoparticle production.

### Solid lipid nanoparticles preparation

Solid lipid nanoparticles were prepared from ethanolic extracts using the hot homogenization method and subsequently reduced in size using an ultrasonic device (Hielscher UP400)^23,25^. The PEE was introduced in its molten state, followed by the addition of an aqueous surfactant solution (Tween-80/Tween-20) at the same temperature, and the mixing process was performed using an ultrasonic probe. The resulting Propolis nano-emulsion underwent a phase transition and transformed into solid lipid nanoparticles (SLNs) due to the thermal shock induced by cold water.

Preliminary experiments showed that the type and concentration of surfactant were effective factors in determining the particle size of SLNs.

To determine the optimal conditions of the system, the optimization section of the Design Expert software was utilized. Tween-20 and Tween-80 were selected as the surfactants, and the surfactant to propolis ratio was adjusted to 25–55% after conducting preliminary experiments. In the following, we also considered the smallest PSLN size produced as the desired and optimal outcome of the software.

Finally, the optimal sample was prepared again according to the optimal conditions specified by the software and evaluated for characterization.

### Characterization

#### Particle size analysis

The particle size of PSLNs was determined by a particle size analyzer (Cordouan Technologies—VASCO NP size analyzer) using the well-known technique of Dynamic Light Scattering (DLS), at ambient temperature.

#### UV–Vis spectroscopy

A small amount of PEE and PSLN were dissolved in DMSO, and their absorption spectra were measured using the Optizon 3230 UV spectrometer (Germany) at wavelengths ranging from 200 to 800 nm.

#### FTIR analysis

Fourier-transform infrared spectroscopy is one of the most widely used methods for identifying the quality of various molecules and for identifying the functional groups present in the structure of a substance. PEE, PSLN, and propolis samples were prepared using potassium bromide (KBr). The spectra of the samples were recorded using the Nicolet Avatar (AVATAR 370, USA) spectrometer, with a range of 400–4000 cm^-1^.

#### XRD analysis

To investigate the crystalline structure of PSLN, we used an X-ray diffractometer (GNR Explorer) within the range of 6° ≤ 2*θ* ≤ 60°.

#### Zeta potential

The zeta potential plays a crucial role in determining the stability of colloid materials. The Zeta compact device (CAD, France) was used to assess the stability of the optimized PSLN at room temperature and pH 7.4.

#### TEM analysis

The morphological characteristics and size distribution of the optimized PSLN were examined using a transmission electron microscope (912 AB LEO, Germany) with a negative staining method involving 2% (w/v) phosphotungstic acid.

#### HPLC analysis

High-performance liquid chromatography was utilized to identify the type and quantity of active ingredients present in the samples. The compounds were identified using an HPLC device equipped with a 46 × 25 cm, C8 reverse phase column (Macherey–Nagel GmbH & Co., Duren, Germany). A mobile phase consisting of a 50:50 v/v solution of acetonitrile and water was used. The isocratic flow rate was adjusted to 1 mL/min for 15 min. To determine the active ingredient content of the samples, we utilized pure salicylic acid, parahydroxybenzoic acid, and vanillic acid as standard compounds.

A control sample of 20 μL DMSO was injected into the device. 20 μL of the standards, PEE, and PSLN samples were injected into the column, and the absorbance was measured at a wavelength of 280 nm. The amount of compounds was determined by comparing the retention time and peak area of the samples.

#### Total phenolic assay

Total phenolic compounds were measured using the Folin-Ciocalteu colorimetric method, which is the most commonly used method for this purpose. The method involves the reduction of the Folin reagent by phenolic compounds in an alkaline environment, resulting in the formation of a blue complex that shows maximum absorption at 760 nm. The total phenol content is determined using the standard curve of gallic acid^26^.

#### Total flavonoid assay

The colorimetric method using aluminum chloride was employed to determine the total flavonoid content. This is the most commonly used method for measuring flavonoids. The method is based on the formation of stable acidic complexes between aluminum chloride and flavonoids that contain carbon 4, keto, or carbon 3 or 5 hydroxyl groups in the A or B rings. These complexes exhibit the highest absorbance at 415 nm^27^.

### The biological properties

#### Antioxidant activity assay

The antioxidant activity of compounds was measured using the DPPH and ABTS radical scavenging activity evaluation methods^28^.

#### Antimicrobial properties

The antimicrobial properties of PEE and PSLN were evaluated using microdilution^29^ and disk diffusion methods.

To investigate the antimicrobial properties of PEE, we prepared a stock solution with a concentration of 8 mg/mL in 8% v/v DMSO. To assess the stability of the PSLN in deionized water and its antimicrobial properties, we used a stock solution prepared at a concentration of 8 mg/mL in deionized water.

#### Evaluation of cytotoxicity

The MTT assay was used to assess the cytotoxicity of the compounds. This method is widely used for measuring the metabolic activity of cells. The A549 cell line was used in this study. A549 is a malignant cell line derived from human lung epithelial carcinoma.

The basis of this method is the ability of the mitochondrial enzyme sucrose dehydrogenase in living cells to convert the yellow, water-soluble MTT salt into water-insoluble formaldehyde crystals^30^. The human dermal fibroblast (HDF) cell line was used to evaluate the biocompatibility of the optimized PSLNs^31^. The cells were purchased from the Pasteur Institute (Tehran, Iran).

#### Statistical test

All experiments in this study were repeated three times, and the data were expressed as the standard deviation. The IC_50_ samples were calculated using the Prism software.

## Results and discussions

### The PEE preparation

The PEE prepared in this study was dark brown in color and had a scent similar to crude propolis. The initial amount of propolis was 2.5 g, and only 0.6 g remained on the filter paper after filtration. The extraction efficiency was approximately 76%. The results of the study conducted by Paviani et al.^24^ on propolis extraction yield demonstrated that extracting raw green propolis using different solvents showed high extraction yields, particularly when ethanol was used as the solvent.

### PSLN preparation from PEE

PSLN nanoparticles were synthesized and found to be relatively stable in deionized water. The size and polydispersity index of the nanoparticles were analyzed using DLS analysis. All the results obtained from the experiments conducted under various conditions were imported into the Design Expert software to determine the optimal synthesized equation. Equation (2) is used in the software to calculate the dimensions of nanoparticles that are synthesized under various conditions, using the given input data.

2 $$ size = + 13989.54103 - \left( {1393.99166 \times {\text{A}}} \right) + \left( {50.97740 \times A^{2} } \right) - \left( {0.80545 \times A^{3} } \right) + \left( {4.65931E^{ - 003} \times A^{4} } \right) $$

In this relationship, (A) represents the percentage ratio of surfactant to lipid. It is worth mentioning that the above equation is valid only within the parameters provided to the software (percentage ratio of surfactant to lipid 25–55%). It is not possible to make definitive comments about points beyond those parameters.

According to the results obtained from the software, it was predicted that the smallest size of PSLNs would be synthesized under the conditions of using Tween 20 as a surfactant and a surfactant-to-lipid ratio of 31%, with dimensions of approximately 73 nm. The experiments were repeated under the provided optimal conditions. An average particle size of 71.97 ± 5 nm was observed during the particle analysis, along with a low polydispersity index of 0.14 and a zeta potential of −10 ± 3 mV, as confirmed by DLS and zeta potential analysis (See Fig. A1 in the supplementary file.) The primary factor contributing to the stability of colloids is the presence of surface charge on the particles. The higher the surface charge of the colloid particles, the more stable the colloid becomes and the longer its particles endure. The magnitude of the zeta potential is related to the surface charge density of the particles. The result of the zeta potential analysis in this study ( − 10 ± 3 mV) is sufficient to prevent the particles in the prepared solution from aggregating and increasing in size over time. The prepared PSLN demonstrates acceptable stability.

#### TEM analysis

**Figure 1:**
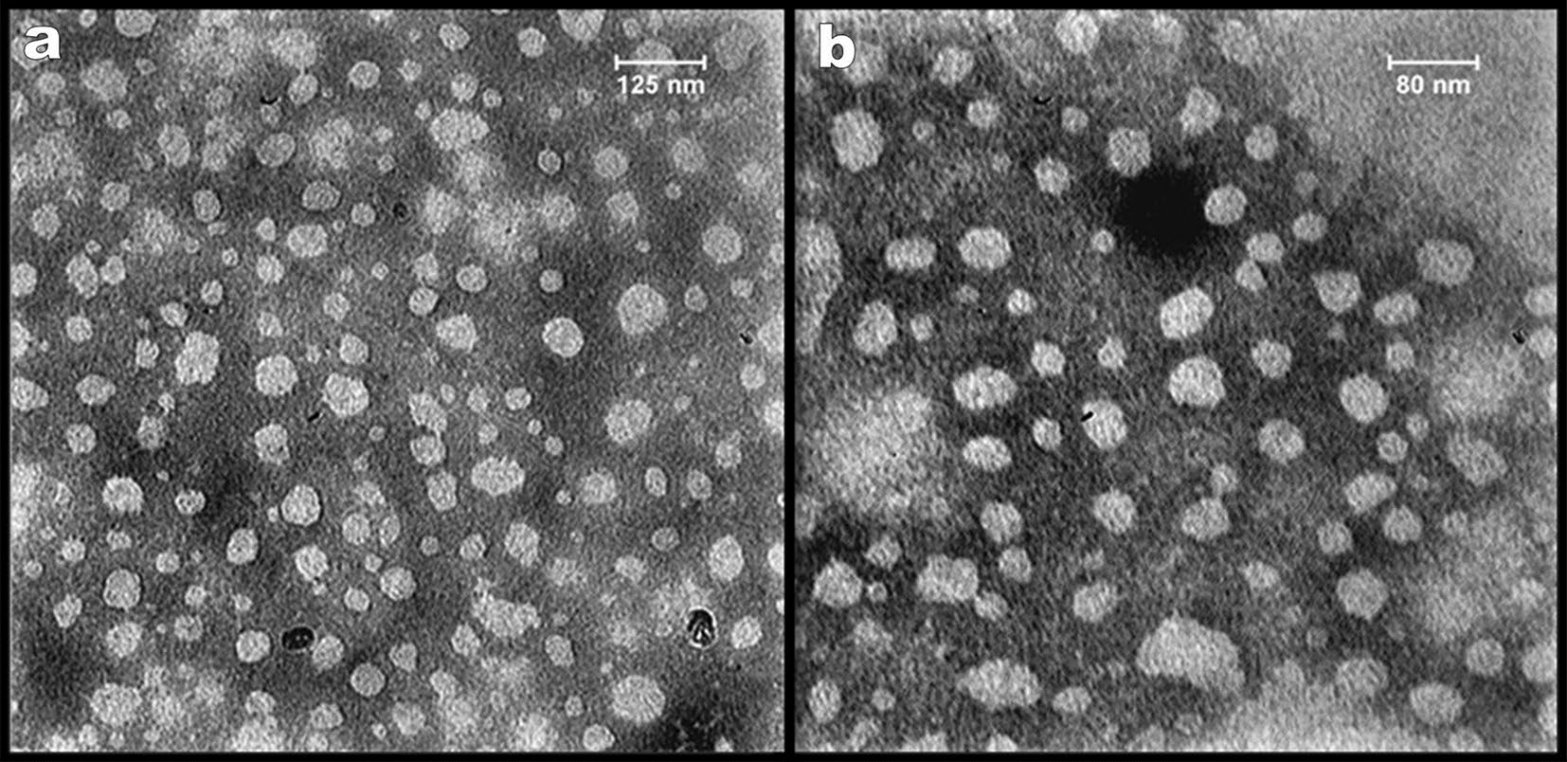
Transmission Microscope image from PSLN in two magnifications. (**a**) Scale-bar length = 125 nm, (**b**) Scale-bar length = 80 nm.

Figure 1 displays the structure of the PSLN that was captured by the TEM microscope. As depicted in the figure, the particle structure is nearly spherical and amorphous. Rosseto et al.^17^ successfully synthesized nanoparticles from propolis, which exhibited an almost spherical and amorphous shape. The average particle size was 57.55 ± 15 nm, calculated using Digimizer software.

#### Fourier-transform infrared spectroscopy

**Figure 2:**
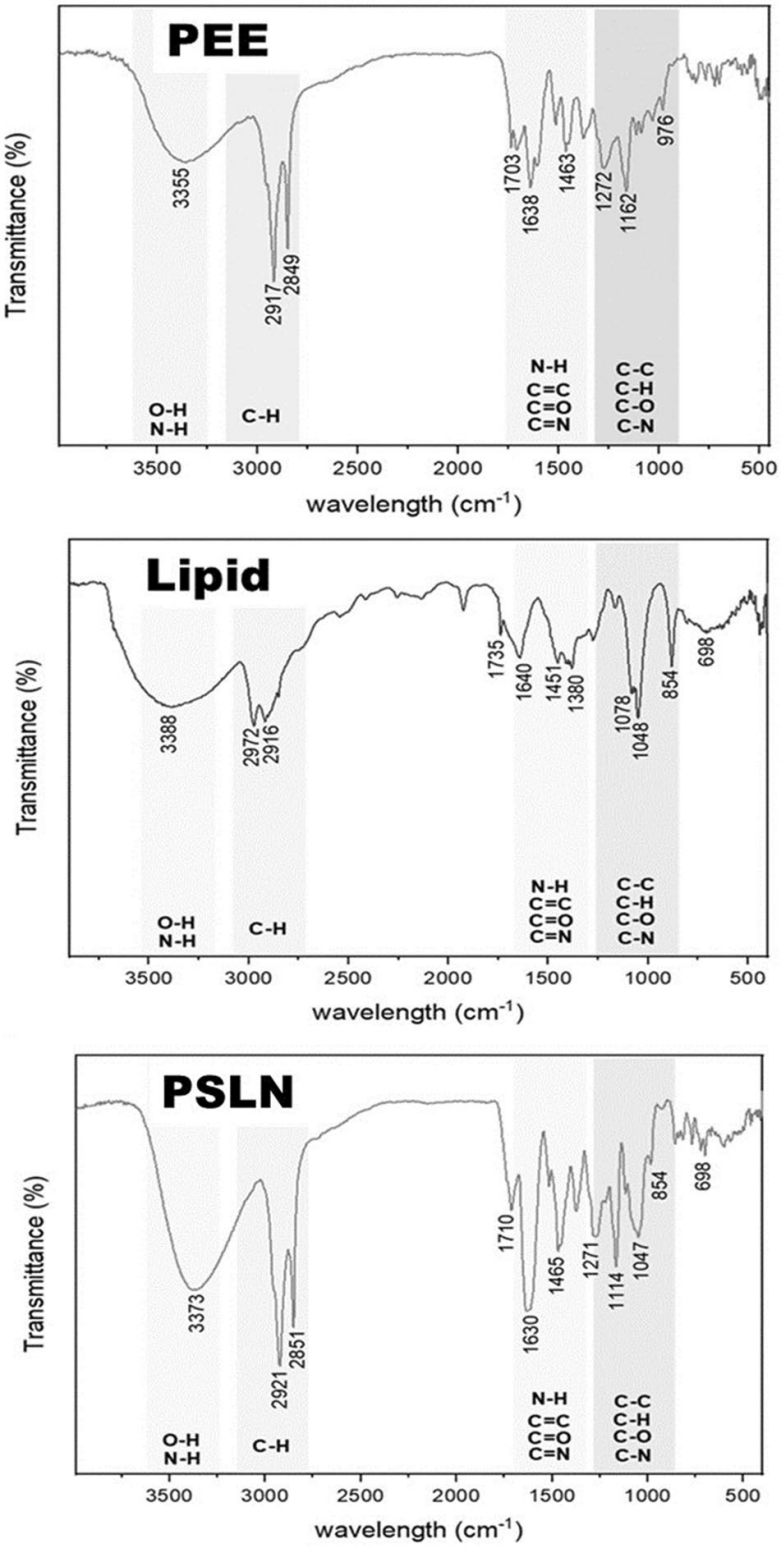
FTIR analysis of PEE, Lipid extracted of PEE and PSLN.

To analyze the functional groups, present in the samples' structures, FTIR analyses were conducted on PEE, PSLN, and the lipid extracted from PEE (as shown in Fig. 2).

The presence of a peak in the 1600–1700 cm^−1^ region is indicative of type I amides. Adsorption peaks between 3200 and 3600 cm^−1^ are related to N–H vibrations in peptide bonds or O–H vibrations in alcoholic and phenolic groups. The absorption peak in the region of 2840 to 3000 cm^−1^ corresponds to the stretching vibrations of methyl (C–H) in aldehyde compositions. The peak at 1640–1690 cm^−1^ corresponds to the stretching vibrations of carbonyl (C=O). The peak at 1020 to 1360 cm^−1^ corresponds to C–N stretching vibrations in aliphatic amines and aromatics. The peak at 1400 to 1600 cm^−1^ corresponds to stretching vibrations of C=C in aromatic rings or N–H vibrations in type II amines. Finally, the peak at 675 to 1000 cm^−1^ corresponds to C–H aromatic compounds^32^.

The presence of all these peaks in all three samples indicates the presence of a wide range of metabolites, especially phenolic and flavonoid compounds. The absorption spectrum in the 1500–1600 cm^-1^ range involves the rearrangement of aromatic rings in phenolic and flavonoid compounds. The presence of a peak at 1630 cm^-1^ corresponds to the stretching vibrations of carbonyl C=O in the structures of flavonoids and lipids^33^. In the research conducted by GG de Lima et al.^34^ and Gatea et al.^35^, they also reported the presence of a peak at 1630 cm^−1^ in the spectrum.

#### X-ray diffraction analysis

**Figure 3:**
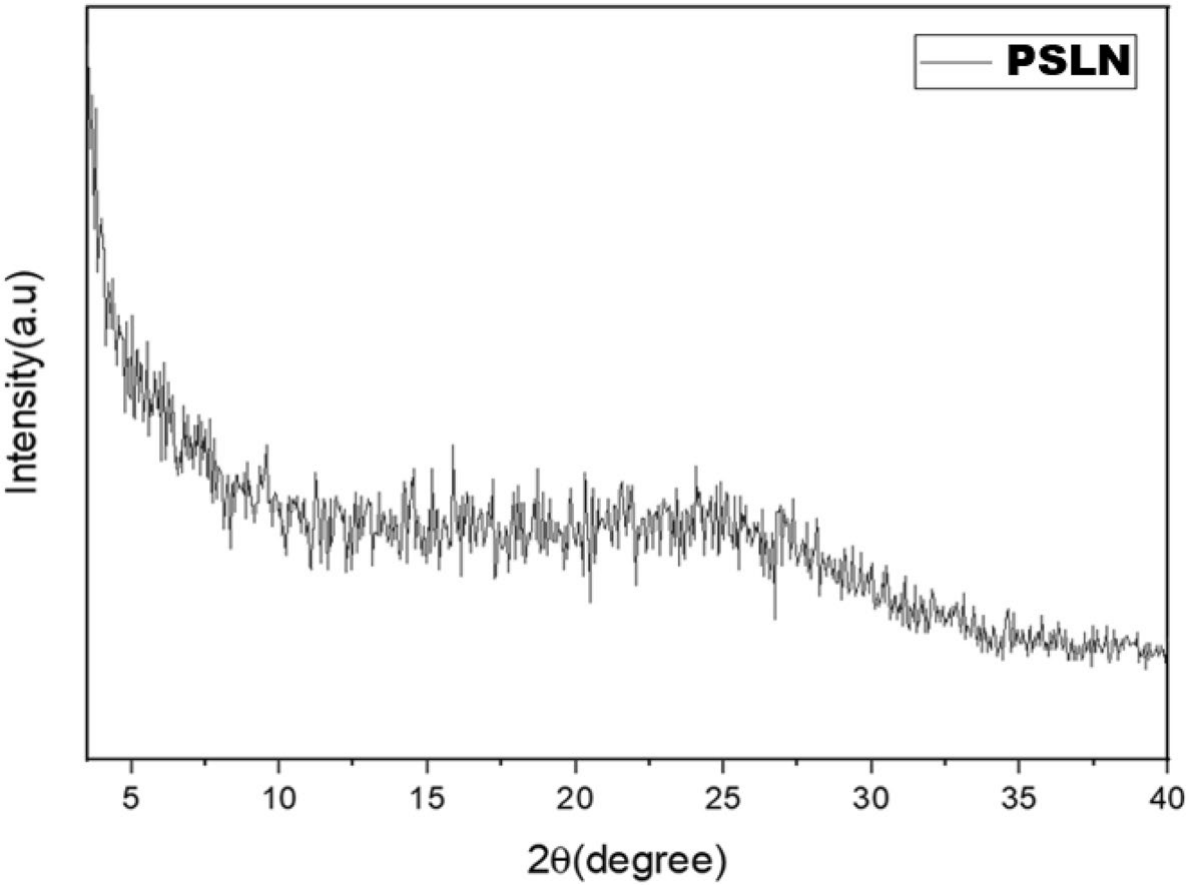
The crystal structure of PSLN was analyzed using the GNR Explorer X-ray diffractometer model within the range of 6° ≤ 2*θ* ≤ 60°.

The X-ray diffraction pattern of PSLN is shown in Fig. 3.

The intensity and type of bond depicted in the figure illustrate the amorphous structure of the PSLN and its lipidic nature, providing evidence for the proper formation of the PSLN structure. Asawahame et al.^36^ produced propolis nanofibers in their research, and the results of their X-ray diffraction analysis confirmed the amorphous structure of propolis in both its regular and nano forms.

### Investigating phenolic and flavonoid compounds

#### UV–Visible spectroscopy

**Figure 4:**
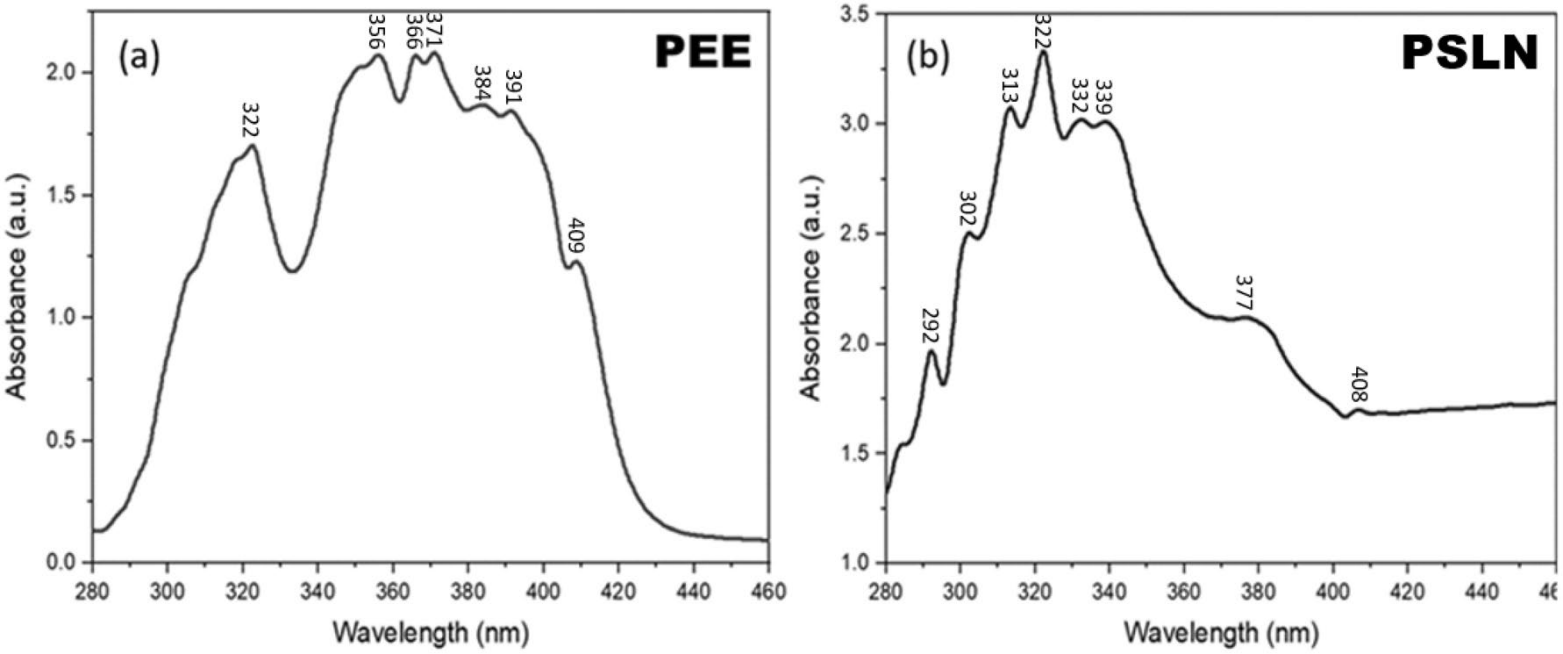
UV–visible spectroscopy of (**a**) PEE and (**b**) PSLN samples. Both samples exhibit a maximum wavelength (λ max) of 322 nm, as determined by the utilization of the Optizon 3230 UV spectrometer.

In Fig. 4, a specific peak at 322 nm is present in both samples, indicating the presence of flavonoid compounds. The presence of this peak, as well as other peaks in the range from 270 to 320 nm, indicates that effective compounds have not been lost during the nano-forming process. Previous studies have also confirmed the peaks of Propolis^37–39^.

#### Identification of compounds using HPLC

The presence of salicylic acid, Para hydroxybenzoic acid, and vanillic acid in PEE and PSLN was investigated by comparing their retention times to those of standard samples.

**Figure 5:**
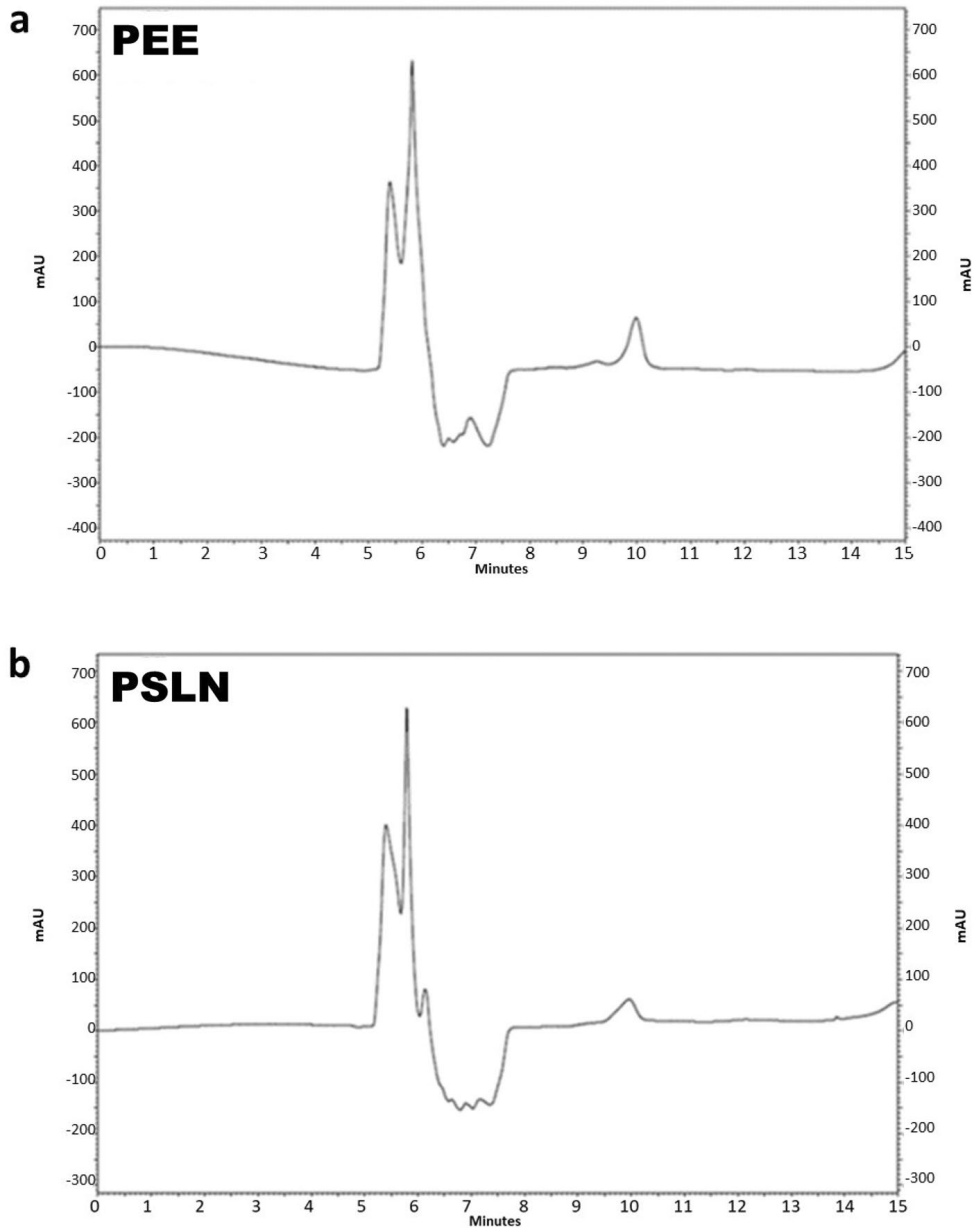
Identification of compounds using HPLC (**a**) PEE, (**b**) PSLN. The amount of compounds in PEE and PSLN was quantified by calculating the area under the graph. The HPLC was manufactured by Macherey–Nagel GmbH & Co. in Düren, Germany.

As depicted in Fig. 5, only the salicylic acid peak was observed in both samples. The standard retention time for salicylic acid was 10.6 min. The peak concentration of salicylic acid was observed in PEE and PSLN samples at 9.98 and 9.8 min, respectively. The amount of salicylic acid in PEE and PSLN was quantified to be 0.003 and 0.001 mg/mL, respectively. AL-Ani et al.^40^, Falcão et al.^41^, and Tykhonov et al.^42^ also identified varying levels of salicylic acid in the PEE through the use of HPLC.

Madan et al.^43^ demonstrated that salicylic acid and its bromine and chlorine derivatives exhibit potent antimicrobial activity against pathogenic *E. coli* bacteria. Vejselova et al.^44^ demonstrated that salicylic acid induces toxicity and inhibits growth in the A549 cell line, ultimately leading to apoptosis. The presence of salicylic acid in PEE and PSLN can be attributed to many of their biological properties due to the various properties of this compound.

#### Determination of total phenols and total flavonoids

The total amount of phenolic compounds was determined using the colorimetric method with Folin-Ciocalteu reagent, based on the concentration of gallic acid equivalents (refer to Fig. A2 in the supplementary file). The total flavonoid content in PEE and PSLN was determined using the colorimetric method with aluminum chloride. The results were expressed in mg of quercetin per gram of sample (see the calibration curve in the appendix file Fig. A3). Table 1 displays the quantity of phenols and flavonoids found in PEE and PSLN at concentrations of 50, 100, and 250 μg/ml.

**Table 1:** The amount of phenols and flavonoids in PEE and PSLN, (*p* < 0.0001). The flavonoid content was quantified using the quercetin standard curve, while the phenol content was determined using the gallic acid standard curve. The concentration of the samples ranged from 50 to 250 µg/ml.

Concentration (µg/ml)	The amount of flavonoids (mg quercetin / gram of sample)		The amount of phenols (mg gallic acid/gram of sample)	
	PEE ± SD	PSLN ± SD	PEE ± SD	PSLN ± SD
50	44.806 ± 0.985	32.667 ± 1.047	264.048 ± 2.354	238.810 ± 0.981
100	81.318 ± 1.985	76.580 ± 1.378	314.524 ± 1.245	304.048 ± 1.257
250	97.829 ± 3.214	90.109 ± 2.321	415.952 ± 0.987	369.762 ± 3.254

According to the results recorded in Table 1, the concentration of phenolic and flavonoid compounds in PEE is significantly higher than that in PSLN (*P* < 0.0001). This difference may be attributed to the elimination of certain phenolic and flavonoid compounds during the nano-forming process. Hafshejani et al.^45^ reported that when comparing propolis collected from different regions of Iran, the propolis collected from Binalood Mountain has the highest amount of phenol and flavonoid compounds.

Tiveron et al.^46^ evaluated the levels of phenols and flavonoids in 78 samples of propolis collected from various regions of Brazil. The highest amounts of phenol and flavonoids were 79.84 mg of gallic acid per gram of sample and 10.3 mg of quercetin per gram of sample in the sample with a concentration of 482.7 µg/mL, respectively. Wali et al.^47^ examined the levels of phenols and flavonoids collected from various regions of Kashmir. The highest levels of phenol and flavonoids were 260 mg of gallic acid/g of sample and 105 mg of quercetin/g of sample in the sample with a concentration of 50,000 µg/mL, respectively. The primary factor contributing to the variation in phenols and flavonoids across regions is the distinct vegetation found in each area. But, in general, the content of phenolic and flavonoid compounds in the propolis sample in this study is significantly higher compared to samples from other regions. On the other hand, due to the similarity of phenolic and flavonoid compounds in the PSLN and the PEE, it can be concluded that the preparation of the nanoparticles largely preserved the main beneficial compounds while removing the resin compounds and other additional substances.

### Antioxidant activity assay

The antioxidant activity of PEE and PSLN was evaluated using the DPPH, and ABTS methods.

The inhibition% of free radicals are presented in Table 2.

**Table 2:** Inhibition% DPPH, ABTS test Results, (*P* > 0.05 for both tests). The color of the DPPH methanolic solution is violet, and it exhibits maximum absorption at a wavelength of 517 nm. ABTS stable cation radical (ABTS^°+^) exhibits a blue-green color and shows maximum absorption at a wavelength of 734 nm. The concentration of the samples ranged from 50 to 250 µg/ml.

Concentration (µg/ml)	DPPH 517 nm		ABTS 734 nm	
	PEE ± SD	PSLN ± SD	PEE ± SD	PSLN ± SD
50	49.409 ± 0.012	56.612 ± 0.012	54.02 ± 0.009	51.09 ± 0.009
100	61.827 ± 0.009	66.695 ± 0.011	82.33 ± 0.022	79.64 ± 0.020
250	79.372 ± 0.022	80.899 ± 0.012	90.94 ± 0.007	88.01 ± 0.007

The results indicate that increasing the concentration of PEE and PSLN enhances the inhibitory power of free radicals. The p-values in both the ABTS and DPPH tests were greater than 0.05. As a result, the PSLNs produced can be used as additives in the food industry due to their antioxidant properties^47^.

Since the structure of PSLN resembles lipid particles, it is evident that the antioxidant performance of PSLN in the ABTS test is weaker compared to that of PEE. ABTS is soluble in both water and organic solvents, whereas DPPH is only soluble in organic solvents. Therefore, PEE has shown better results due to the presence of a greater number of hydrophilic and lipophilic compounds. The difference in ABTS and DPPH test results once again confirms the formation of PSLN nanoparticles.

Table 3 displays the IC_50_ values of PEE and PSLN samples for the reduction of DPPH and ABTS.

**Table 3:** The IC_50_ values of PEE and PSLN samples for reduction of DPPH and ABTS.

Sample	Method	*IC*_*50*_ (mg/ml)	R^2^
PEE	DPPH	0.030 ± 0.00088	0.979
PSLN		0.028 ± 0.00083	0.943
PEE	ABTS	0.024 ± 0.00084	0.920
PSLN		0.028 ± 0.00086	0.938

The variation in IC_50_ values observed in these studies could be attributed to differences in the initial concentration of the extract. The extracts possess antioxidant properties attributed to the presence of phenolic and flavonoid compounds. Therefore, the presence of vegetation and the high concentration of flavonoids and phenols such as salicylic acid^48^ in the extract effectively inhibit free radicals.

Antioxidants are typically categorized into two groups according to their mode of action: preventive and chain-breaking. Propolis' phenolic compounds are classified as chain-breaking antioxidants, which neutralize peroxyl radicals required for chain propagation. Consequently, the chain length is reduced, and the antioxidant's efficacy is established^49^.

In the process of making PSLN from PEE, some of the antioxidant substances in the composition may be lost. But the results of the antioxidant test show that the antioxidant abilities of PSLN and PEE are almost equal. The main point of this discussion is that when materials are synthesized at the nanoscale, their surface-to-volume ratio increases significantly. This leads to enhanced material activity compared to their regular state. On the other hand, nanoparticles disperse more effectively in solution compared to their bulk state due to their small size and surface charge. This increased dispersion increases the chance of contact between the nanoparticle and the free radicals. On the other hand, Barzegar et al.^50^ have conducted studies on the properties of nano-antioxidants. In their research, they have addressed that antioxidant nanoparticles can prevent oxidative stress in the bloodstream.

Wali et al.^47^, Rebiai et al.^51^, Talla et al.^52^, and Moreira et al.^53^ reported that the IC_50_ values for DPPH free radical inhibition in their best samples were 0.076, 0.06, 5.06, and 0.0528 mg/mL, respectively. Miguel et al.^54^ reported that the collected samples from various regions of southern Portugal had the lowest IC_50_ of DPPH and ABTS free radical inhibition at 0.008 and 0.009 mg/mL, respectively.

According to the recorded results of other research, the propolis from Iran's Binalood Mountain range can be classified as suitable for use as an antioxidant. The main achievement of this research is the synthesis of PSLNs that can effectively preserve the antioxidant properties of the propolis ethanolic extract.

### The antimicrobial properties

#### Determination of MIC and MBC

The inhibitory effects of PEE and PSLN on four strains of pathogens were evaluated using the microdilution method. The MIC and MBC values are presented in Table 4.

**Table 4:** The MIC and MBC values of different bacteria. The bacteria evaluated include *E. coli*, *S. aureus*, *B. subtilis*, and *P. aeruginosa*.

Bacteria	MIC (mg/ml)		MBC (mg/ml)	
	PEE	PSLN	PEE	PSLN
*E. coli*	1 ± 0.034	0.5 ± 0.010	1 ± 0.011	1 ± 0.031
*S. aureus*	0.5 ± 0.021	0.25 ± 0.019	0.5 ± 0.016	0.25 ± 0.011
*B. subtilis*	0.125 ± 0.029	0.125 ± 0.008	0.125 ± 0.004	0.125 ± 0.009
*P. aeruginosa*	2 ± 0.031	1 ± 0.011	2 ± 0.029	2 ± 0.026

The MIC and MBC values obtained for the four pathogen strains showed that PSLN had a higher growth inhibitory potency compared to PEE, which may be due to its small size and ability to easily enter the bacterial cell. The highest and lowest antimicrobial effects were observed for *B. subtilis* and *P. aeruginosa*, respectively, when the two substances were tested. PEE and PSLN had a greater impact on Gram-positive bacteria (*S. aureus* and *B. subtilis*) compared to Gram-negative bacteria (*E. coli* and *P. aeruginosa*), possibly due to the structural membrane variations between these bacteria. The presence of LPS in gram-negative bacteria creates a barrier that prevents antimicrobial agents from entering the bacterial cell and hinders the passage of other compounds into the cell.

Almuhayawi^55^ and Tahmasebi et al.^56^, reported the presence of quercetin, pinocembrin, apigenin, and cinnamic acid in propolis. Quercetin has been identified as having the ability to bind to the DNA gyrase of *E*. *coli*, thereby impeding the growth of the bacteria. The introduction of propolis to bacteria results in partial lysis, which leads to changes in their protein composition. Furthermore, the antibacterial properties of pinocembrin and apigenin, which are also found in propolis, have been observed. Notably, propolis contains a significant amount of cinnamic acid, which exhibits toxic effects on various microorganisms. The mechanism of action involves impairing bacterial membranes, inhibiting ATPase activity, preventing biofilm formation, and disrupting bacterial division, ultimately resulting in membrane damage.

Ristivojević et al.^57^ measured the minimum inhibitory concentration (MIC) values of various types of propolis collected from different regions of Serbia. The lowest reported MIC values for *S. aureus* and *B. subtilis* were 0.5 and 0.3 mg/mL, respectively. Zohdi et al.^58^ calculated the minimum inhibitory concentration (MIC) value of the ethanol extract of propolis for various samples of both Gram-positive and Gram-negative bacteria. The lowest MIC values reported for *S. aureus* and *B. subtilis* bacteria were 9 and 4.5 mg/mL, respectively. In the research conducted by Zhang et al.^21^, red propolis microcapsules exhibited an MIC of 0.135–0.271 mg/mL against *S. aureus*.

#### Disk diffusion method

Table 5 displays the diameter of the inhibition zone for four pathogens when exposed to varying concentrations of PEE and PSLN.

**Table 5:** The diameter of the inhibition zone of four pathogens (*E. coli, S. aureus, B. subtilis,* and *P. aeruginosa*) in the presence of different concentrations of PEE and PSLN. Amp and Van were used as control samples.

Bacteria	Zone of inhibition (mm)							
	PEE			PSLN			Amp*	Van**
Concentration(µg/ml)	0.5	1	2	0.5	1	2	20(µg)	10(µg)
*E. coli*	19.25	25.62	28.12	20.24	25.831	29	41.97	49.42
*S. aureus*	18	19.48	27.25	21.24	26.08	35.24	40	46.91
*B. subtilis*	25.01	27.03	31.045	25.65	39.77	42.21	41.34	48.7
*P. aeruginosa*	12.45	13	16.75	15.31	16.39	16.75	30	32.35

*Ampicillin antibiotic as a control sample.

**Vancomycin antibiotic as a control sample.

The highest and lowest diameters were related to *B. subtilis* and *P. aeruginosa*, respectively, which is consistent with microdilution results. The diameter of the inhibition zone for the PSLN sample was significantly greater than that of the PEE sample at concentrations of 1 and 2 mg/mL (*P* < 0.0001). This difference may be attributed to the solubility of the PSLN sample and its better dispersion in the agar medium. No significant difference was observed in the diameter of the inhibition zone in other concentrations.

Gonsales et al.^59^ examined the diameter of the inhibition zone of PEE collected from various regions of Brazil against *E. coli* and *S. aureus* bacteria. No inhibition zone was observed for *E. coli* in their studies. The largest diameter was 12 mm, which was associated with *S. aureus*. Abduh et al.^60^ analyzed the growth inhibitory effects of various propolis extracts. The inhibition growth zone of *S. aureus* in their research ranged from 6.58 ± 0.04 to 9.70 ± 0.7 mm.

The diameter of the inhibition zone in the presence of varying concentrations of PEE and PSLN was significantly greater than in previous studies. This demonstrates the superior antimicrobial properties of the samples produced in this study.

#### Study of structural changes of E. coli in the presence of PEE and PSLN

To investigate the destruction of the bacterial cell wall, we performed AFM imaging after exposing the cells to 0.5 mg/mL of PEE and PSLN, and after the incubation process.

**Figure 6:**
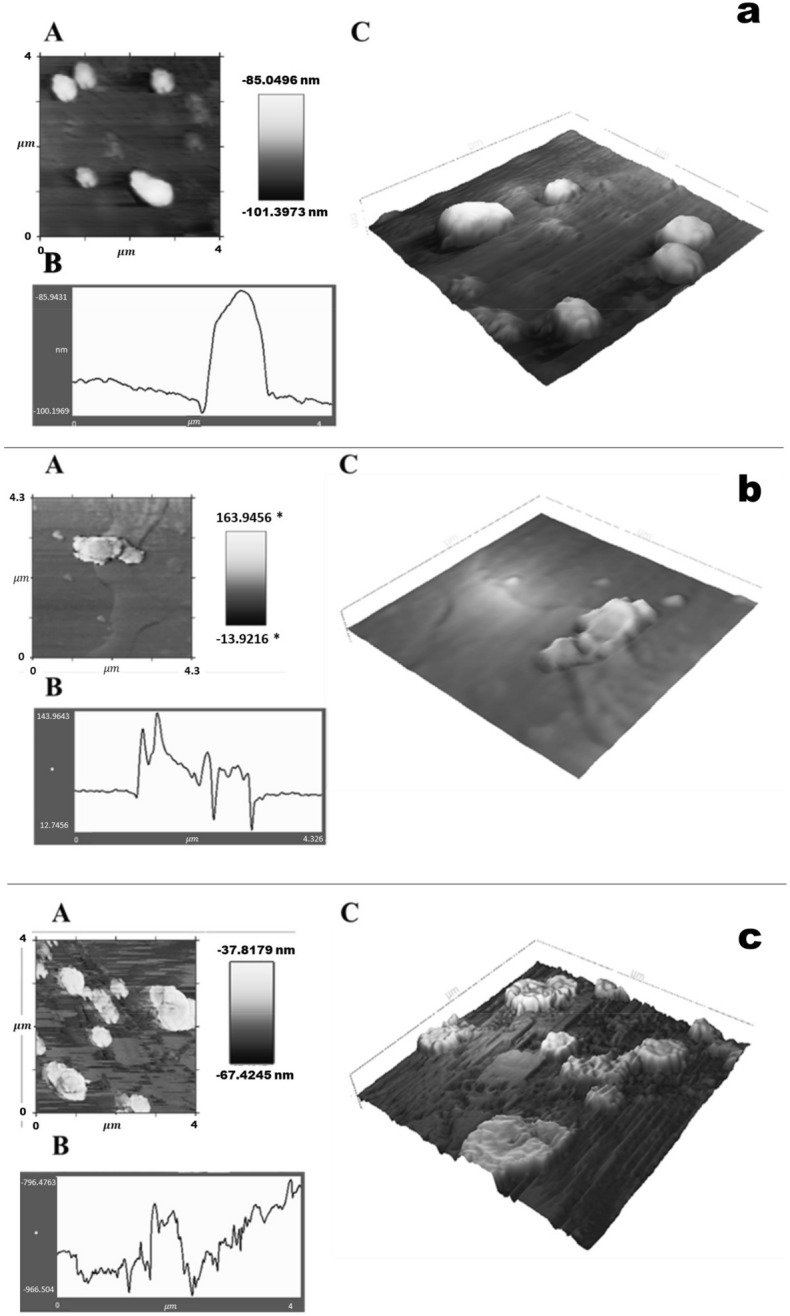
AFM images of the bacterial cell wall. A: 2D image, B: surface roughness, C: 3D image. (**a**) AFM image of *E. Coli* bacteria in the absence of PEE and PSLN. (**b**) AFM image of *E. Coli* bacteria in the presence of 0.5 mg/mL of PEE. (**c**) AFM image of *E. coli* bacteria in the presence of 0.5 mg/mL of PSLN. Imaging by the AFM model Multimode Full Plus A/101.

As shown in Fig. 6a, the bacterial cell wall appears flat in the absence of nanoparticles. After being exposed to PEE and PSLN, these materials bind to the cell wall of the bacterium, causing damage to the cell membrane (Fig. 6b,c). Because the nanoparticles are about 57 ± 15 nm in size, they can penetrate and act on the bacterial cell wall.

#### Cytotoxicity study of PEE and PSLN

**Figure 7:**
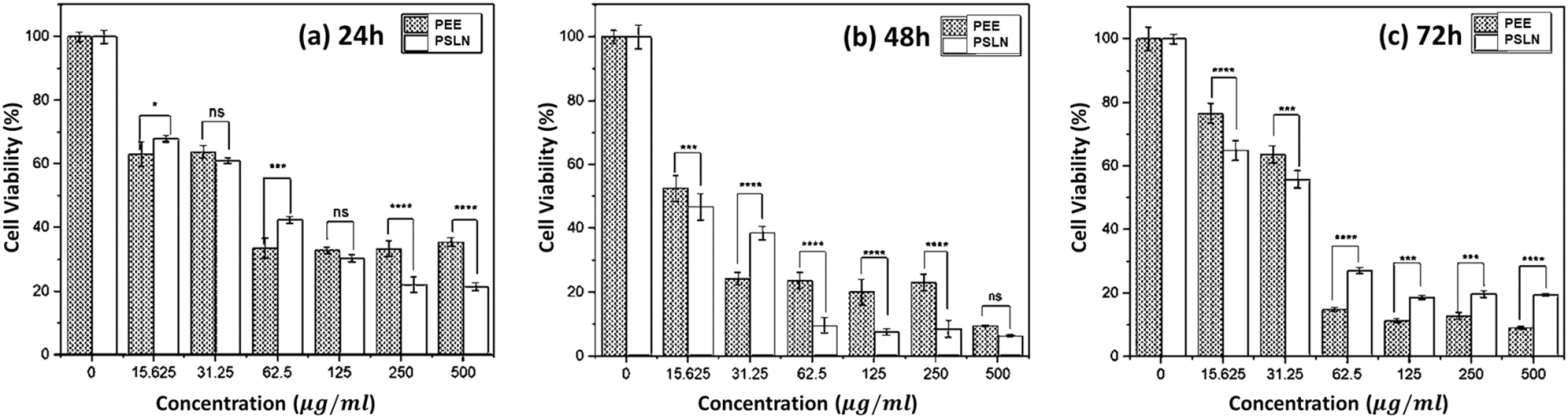
The cell viability percentage of A549 cells was measured after treatment with different concentrations of PEE and PSLN for (**a**) 24 h, (**b**) 48 h, and (**c**) 72 h. Generated using GraphPad Prism version 7 software. (****p < 0.0001, ***p = 0.001, ns = *p* > 0.05).

The cytotoxic effects of PEE and PSLN were evaluated on the A-549 cell line using concentrations of 15.625, 31.25, 62.5, 125, 250, 500, and 1000 μg/mL at three different time intervals (24, 48, and 72 h) using the MTT method. Figure 7 displays the percentage of viable A-549 cells following treatment with varying concentrations of PEE and PSLN at 24, 48, and 72-h intervals.

Based on the results, both PEE and PSLN exhibited comparable cytotoxicity to this cell line. The percentage of live cells decreased as the concentration of both substances increased. The percentage of live cells decreased after 24, 48, and 72 h, and the concentration of PEE at 15.625 μg/mL resulted in a decrease of 63.12%, 62.12%, and 52.54%, respectively. For PSLN, these values were 67.99%, 64.91%, and 67.99%, respectively. The results indicate that the cytotoxic effects of the two substances are dependent on both concentration and time. As the concentration in each sample increased, the percentage of live cells decreased. There was no significant difference in the IC_50_ values between the two samples following 24, 48, and 72-h treatments. However, when comparing the percentage of live cells, significant differences were observed in certain concentrations (Table 6).

**Table 6:** The IC_50_ values of PEE and PSLN were evaluated at concentrations of 15.625, 31.25, 62.5, 125, 250, 500, and 1000 µg/ml (the average results are reported in the table below) on the A-549 cell line over three time periods: 24, 48, and 72 h. It has been evaluated using the MTT method.

Propolis	A549 Cell line IC_50_(mg/ml)		
	24 h	48 h	72 h
PEE	0.0468 ± 0.0009	0.0319 ± 0.0061	0.0150 ± 0.0004
PSLN	0.0451 ± 0.0065	0.0348 ± 0.0022	0.0102 ± 0.0012

Khacha-Ananda et al.^61^ reported the IC_50_ values of PEE on the A549 cell line after 24, 48, and 72 h of treatment as 93.99, 81.89, and 76.02 μg/mL, respectively. Frion-Herrera et al.^62^ examined the cytotoxic effects of PEE collected from various regions of Brazil on the A549 cell line. The results of their study showed that the effect of PEE on this cell line follows a concentration-dependent pattern. They reported an IC_50_ of 69.17 μg/mL for PEE after 72 h of treatment. Demir et al.^63^ reported an IC_50_ value of 31.07 μg/mL for PEE on the cell line after 72 h of treatment. Hanafy et al.^64^ fabricated propolis nanoparticles. The IC_50_ values were reported as 214.3 µg/mL after 24 h and 132.4 µg/mL after 48 h.

The cytotoxic power of PEE and PSLN in this research has been significantly higher than in other studies conducted on this cell line. This may be attributed to the high percentage of flavonoids and phenols like salicylic acid (confirmed using HPLC) found in the PEE and PSLN, respectively, in this study. These compounds can induce apoptosis in this cell line. On the other hand, the results of the biocompatibility test of PSLNs using the MTT test demonstrated that PSLNs did not have a significant impact on the viability of human dermal fibroblasts (HDF) cells (*p* < 0.01).

In the section on zeta potential analysis, it was already mentioned that this analysis was performed at a pH of 7.4, which is the pH of blood, in order to verify the stability of nanoparticles in the bloodstream. According to the remarkable properties of nanoparticles discussed in the study of cytotoxicity and nanoparticle stability, the PSLN synthesized in this research can be an effective biological drug for treating lung cancer or a suitable drug carrier for this type of cancer.

## Conclusion

In this study, an ethanolic extract of propolis (PEE) was prepared from crude propolis collected from Binalood Mountain in Iran. Solid lipid nanoparticles (SLNs) were produced using lipid compounds extracted from PEE. The optimization process was performed using Design Expert software. By utilizing the optimized formula for synthesizing propolis solid lipid nanoparticles (PSLNs), we have successfully generated PSLNs within the size range of 50–60 nm. To verify the formation of PSLN and evaluate its properties in comparison to PEE, several characterization methods were employed. These methods included dynamic light scattering (DLS), zeta potential analysis, UV–visible spectroscopy, Fourier-transform infrared spectroscopy (FTIR), X-ray diffraction (XRD), high-performance liquid chromatography (HPLC), and transmission electron microscopy (TEM) analyses. TEM, XRD, and DLS analyses confirmed the formation of amorphous PSLNs with a spherical morphology, ranging from 50 to 60 nm. The results obtained from zeta potential analysis indicate that these PSLNs exhibit good stability and dispersity in an aqueous medium at a pH of 7.4. UV–Visible spectroscopy, FTIR, and HPLC analyses confirmed the presence of phenolic and flavonoid compounds in PEE and optimized PSLN.

After confirming the properties of PSLN, various biological tests were conducted to evaluate the optimized PSLN's antioxidant, antibacterial, and anticancer properties. DPPH and ABTS assays demonstrated that PEE and PSLN possess significant antioxidant properties. The IC_50_ of PSLN in DPPH and ABTS tests was approximately 0.028 mg/mL and 0.028 mg/mL, respectively.

Both PEE and PSLN exhibited stronger inhibitory effects against various bacterial strains compared to previous studies. MIC, MBC, and disc diffusion tests were used to evaluate the antibacterial properties. PEE and PSLN had a greater impact on Gram-positive bacteria, such as *S. aureus* and *B. subtilis*. The results of the Minimum Inhibitory Concentration (MIC), Minimum Bactericidal Concentration (MBC), and disc diffusion tests of PSLN on *S. aureus* were 0.025 mg/mL, 0.025 mg/mL, and 21.24 mm, respectively. On the other hand, the results for *B. subtilis* were 0.125 mg/mL, 0.125 mg/mL, and 25.65 mm, respectively.

The anticancer test demonstrated that PSLN had a concentration- and time-dependent inhibitory effect on the A-549 cell line. The IC_50_ results for PSLN after 24, 48, and 72 h were 0.045, 0.034, and 0.010 mg/mL, respectively. These results demonstrate the remarkable anticancer properties of optimized PSLN. The synthesized PSLNs have potential applications in the manufacturing of various nano-products, including pharmaceuticals, medications, cosmetics, food, and packaging.

### Supplementary Information

Supplementary Information.

## Data Availability

The datasets used and/or analysed during the current study available from the corresponding author on reasonable request.
