# Supplementary material for: Synthesis of optimized propolis solid lipid nanoparticles with desirable antimicrobial, antioxidant, and anti-cancer properties
Source: Sci Rep. 2023 Oct 25;13:18290. doi: 10.1038/s41598-023-45768-y (PMC10600131; doi:10.1038/s41598-023-45768-y)
Supplement: Supplementary file 1 — Supplementary Information. [file 41598_2023_45768_MOESM1_ESM.docx]

**Appendix**


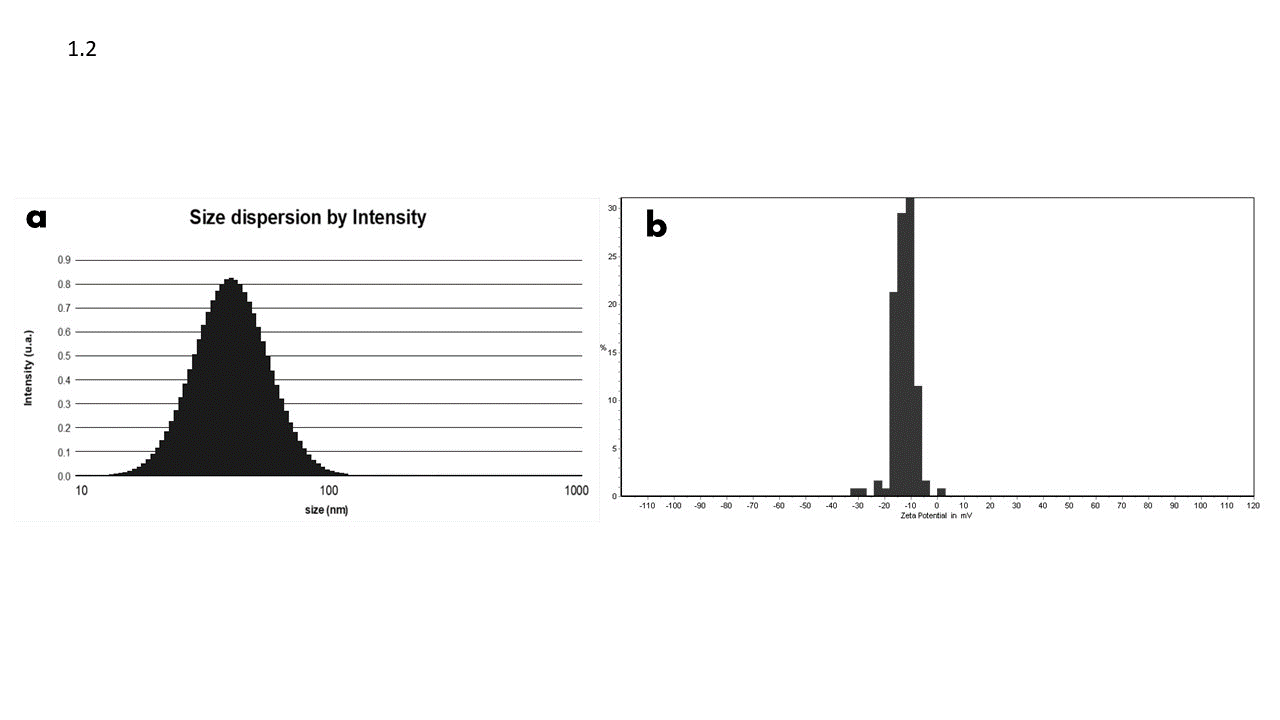


**Figure A1: The results of the dynamic light scattering (DLS) and zeta potential analysis of PSLN.**


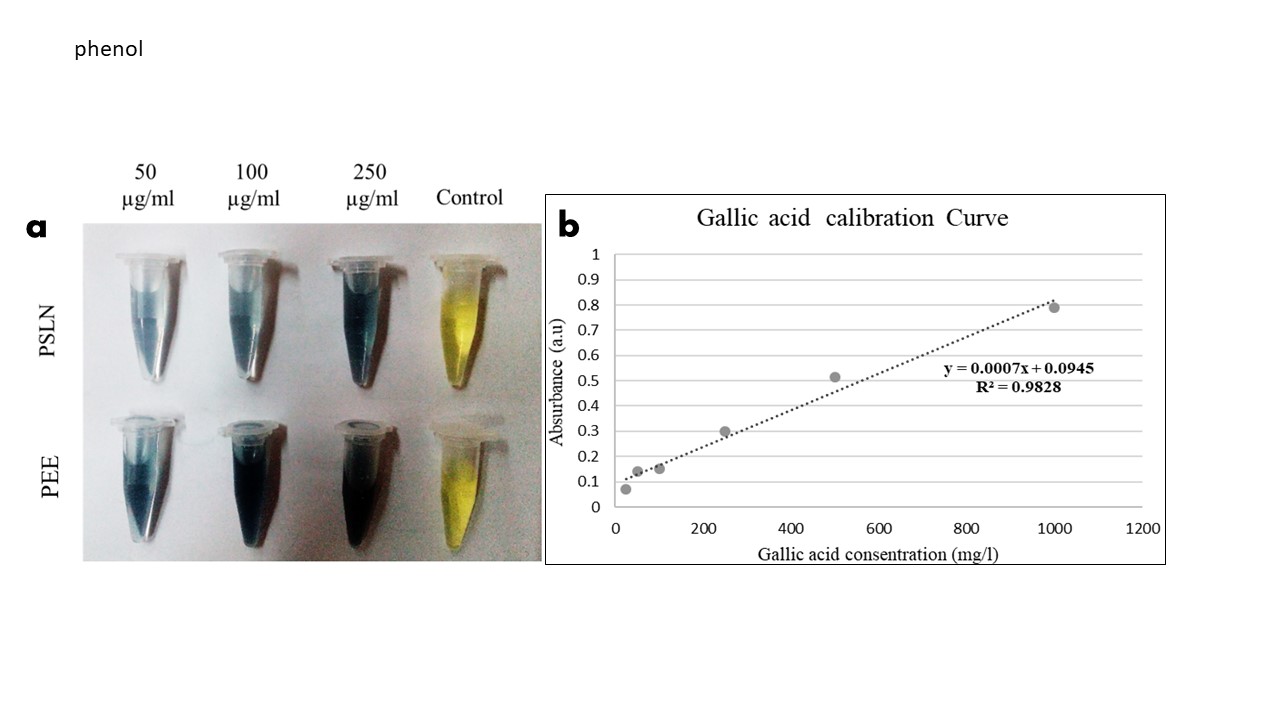


**Figure A2: Measuring the amount of phenolic compounds; (a) Determination of total phenol by Folin Ciocalto method; (b) Gallic acid calibration curve.**


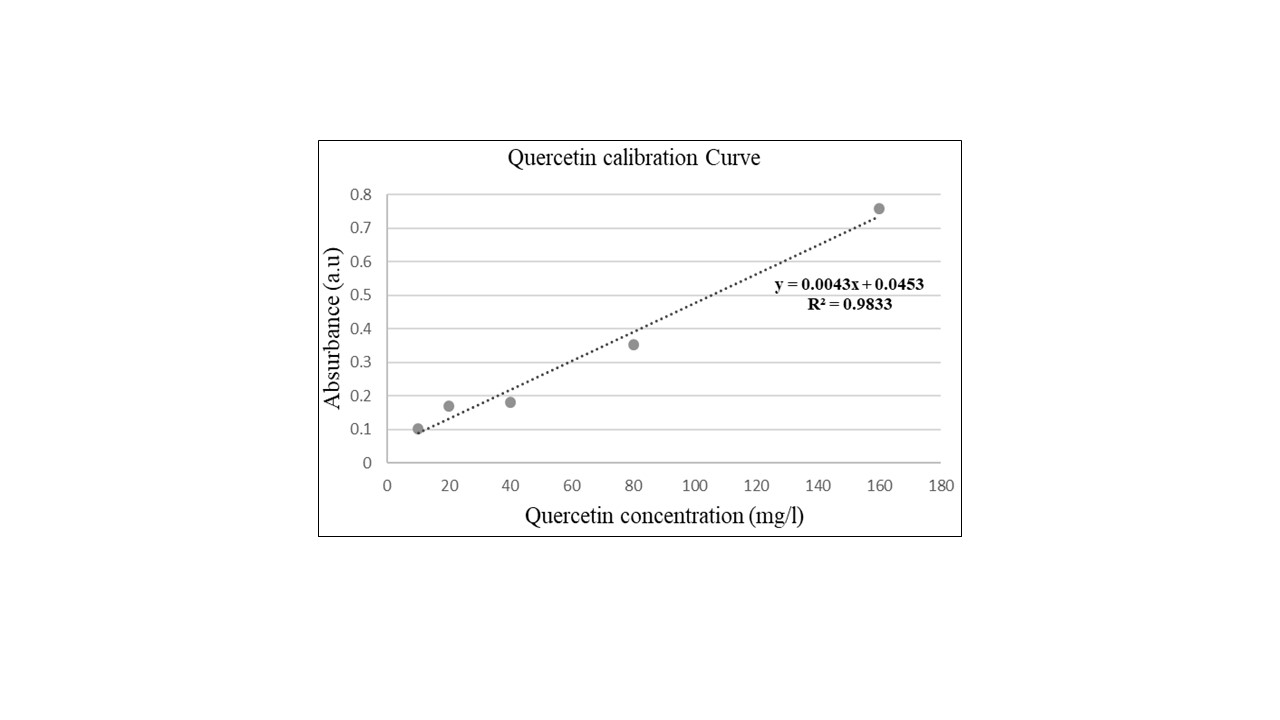


**Figure A3: Calibration Curve for Quercetin.**


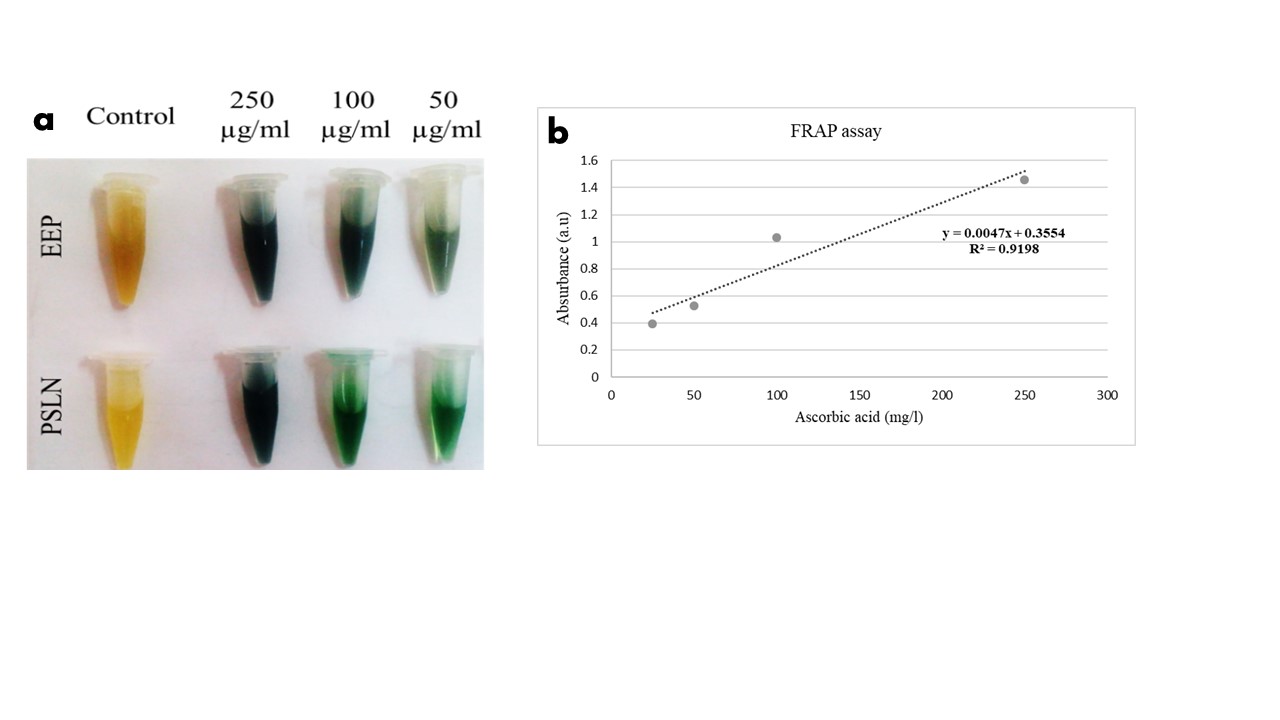


**Figure A4: (a) FRAP test results for PEE and PSLN samples; (b) calibration curve for ascorbic** **acid.**


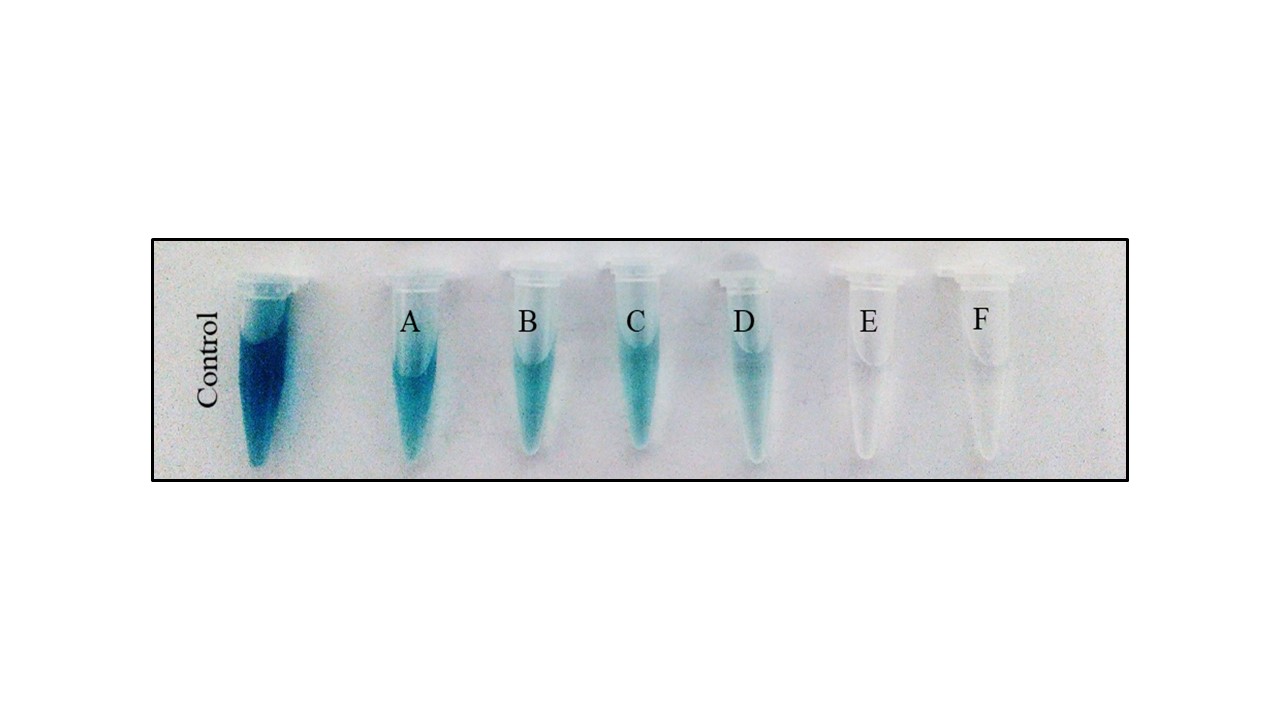


**Figure A5: Regeneration of ABTS in the presence of varying concentrations of PEE. Concentrations ranging from 6.25 to 200 µg/ml are represented by the values A to F, respectively.**


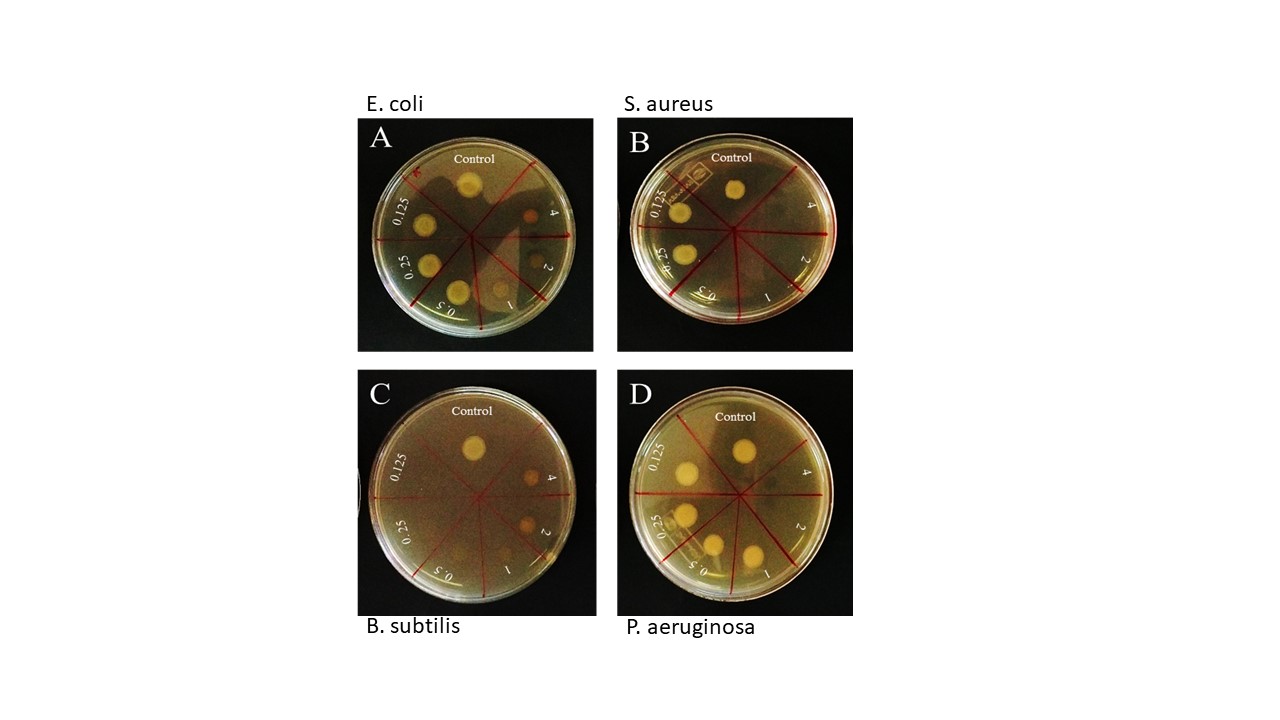


**Figure A6: MBC test images for PEE after 24 hours in a greenhouse: Images A, B, C, and D correspond to E. coli, S. aureus, B. subtilis, and P. aeruginosa bacteria, respectively. The control cells indicate the presence of bacteria alone and in the absence of antimicrobial substances. The numbers on the plate indicate the concentration of the samples in mg/ml.**


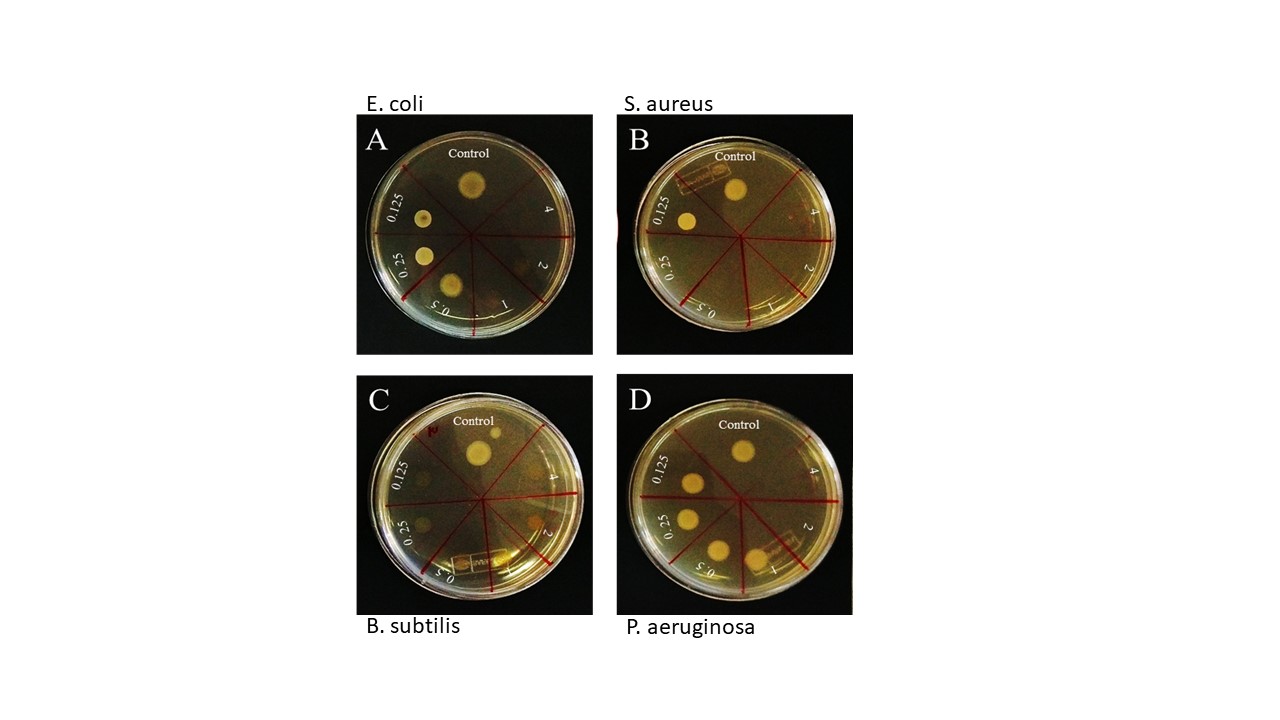


**Figure A7: MBC test images for PSLN after 24 hours. Images A, B, C, and D correspond to E. coli, S. aureus, B. subtilis, and P. aeruginosa bacteria, respectively. The control cells indicate the presence of bacteria in isolation, without the presence of antimicrobial substances. The numbers on the plate indicate the concentration of the samples in milligrams per milliliter (mg/mL).**


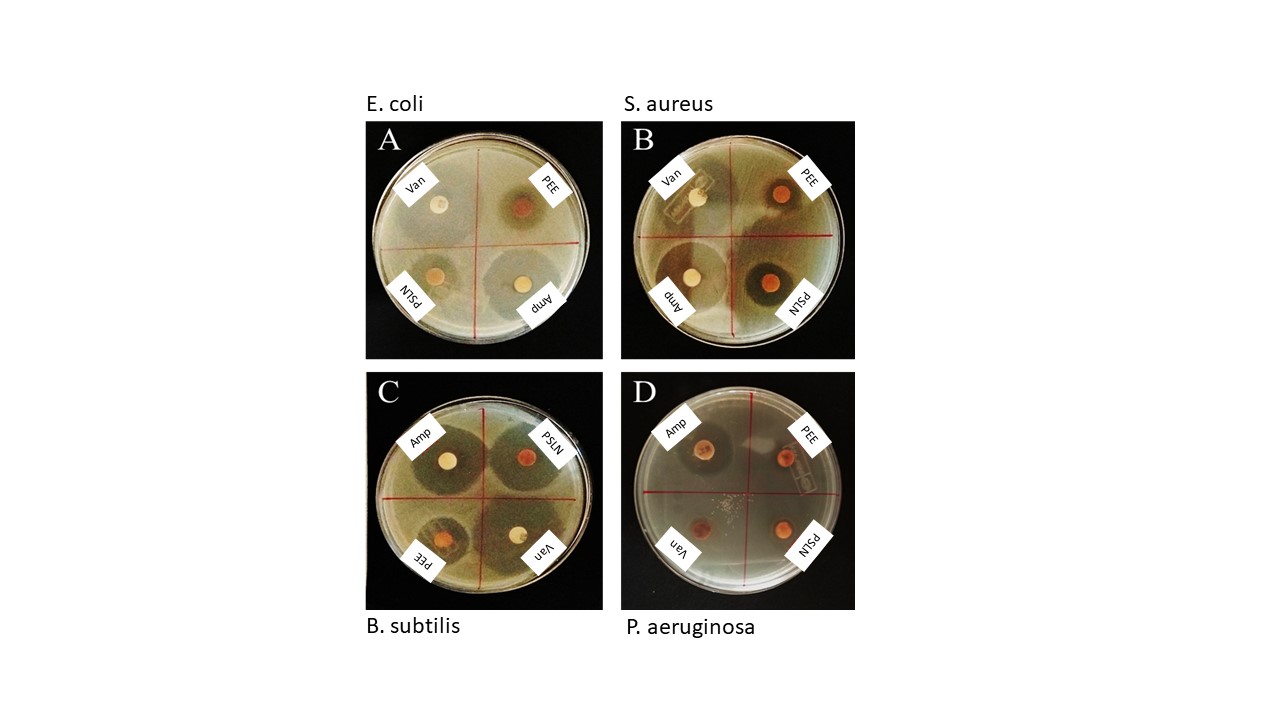


**Figure A8: The non-growth halo test images for EEP and PSLN at a concentration of 1mg/ml after 24 hours are as follows: Image A to D are related to E. coli, S. aureus, B. subtilis, and P. aeruginosa bacteria. Amp and Van are antibiotic discs containing 20 µg of ampicillin and 10 µg of vancomycin, respectively.**
